# Phase I/II study of S-1 combined with irinotecan for metastatic advanced gastric cancer

**DOI:** 10.1038/sj.bjc.6603072

**Published:** 2006-03-28

**Authors:** M Inokuchi, T Yamashita, H Yamada, K Kojima, W Ichikawa, Z Nihei, T Kawano, K Sugihara

**Affiliations:** 1 Department of Esophagogastric Surgery, Tokyo Medical and Dental University, 1-5-45, Yushima, Bunkyo, Tokyo 113-8519, Japan; 2 Mutsugi Clinic, 1-4-14, Mutsugi, Adachi-ku, Tokyo 121-0052, Japan; 3 Department of Digestive and General Surgery, Saitama Medical School, 38, Moro-Hongo, Moroyama-cho, Iruma-gun, Saitama 350-0495, Japan; 4 Tojun Hospital 4-3-4, Hitotsuya, Adachi, Tokyo 121-0075, Japan; 5 Department of Surgical Oncology, Tokyo Medical and Dental University, 1-5-45, Yushima, Bunkyo, Tokyo 113-8519, Japan

**Keywords:** S-1, irinotecan, combination chemotherapy, advanced gastric cancer

## Abstract

A dose-escalation study of irinotecan (CPT-11) combined with S-1, an oral dihydropyrimidine dehydrogenase inhibitory fluoropyrimidine, was performed to determine the maximum-tolerated dose (MTD), recommended dose (RD), dose-limiting toxicities (DLTs), and objective response rate (RR) in advanced gastric cancer (AGC). S-1 was administered orally at 80 mg m^−2^ day^−1^ from day 1 to 14 of a 28-day cycle and CPT-11 was given intravenously on day 1 and 8 at an initial dose of 70 mg m^−2^ day^−1^, stepping up to 100 mg m^−2^. The treatment was repeated every 4 weeks, unless disease progression was observed. In the phase I portion, the MTD of CPT-11 was presumed to be 100 mg m^−2^, because 66.6% of patients (two of three) developed DLTs. All three patients at the initial RD of CPT-11 (90 mg m^−2^) experienced grade 4 haematological or grade 3 nonhaematological toxicities at second course, followed by the dose reduction of CPT-11 from the third course. Considering safety and the ability to continue treatment, the final RD was determined to be 80 mg m^−2^. In the phase II portion, 42 patients including seven patients in the final RD phase I portion were evaluated. The median treatment course was five (range: 1–13). The incidences of severe (grade 3–4) haematological and nonhaematological toxicities were 19 and 10%, respectively, but all were manageable. The RR was 62% (26 of 42, 95% confidence interval: 47.2–76.6%), and the median survival time was 444 days. Our phase I/II trial showed S-1 combined with CPT-11 is effective for AGC and is well tolerated, with acceptable toxicity.

Unresectable advanced or recurrent gastric cancer still has a poor prognosis despite chemotherapy. Many randomised phase III studies of combination chemotherapy for unresectable advanced gastric cancer (AGC) resulted in median survival times (MSTs) of the 5–9.6 months and overall response rates (RRs) of 9–46% in Western and Asian countries (Webb *et al*, 1997; Icli *et al*, 1998; Vanhoefer *et al*, 2000; Ross *et al*, 2002; Ohtsu *et al*, 2003). The significant survival advantage of 5-fluorouracil (5-FU)-based chemotherapy for AGC has been demonstrated, compared with best supportive care (Murad *et al*, 1993; Pyrhonen *et al*, 1995; Glimelius *et al*, 1997); however, a standard regimen for AGC has not yet been established. New anticancer drugs, such as oral fluoropyrimidines, taxanes, and irinotecan hydrochloride (CPT-11), have been developed and their antitumour effects against gastric cancer and good feasibility have been demonstrated (Futatsuki *et al*, 1994; Ajani *et al*, 1998; Sakata *et al*, 1998; Graziano *et al*, 2000; Koizumi *et al*, 2000).

S-1 is an oral fluorinated pyrimidine that combines tegafur with two 5-FU-modulating substances, 5-chloro-2,4-dihydroxy pyridine and potassium oxonate, in a molar ratio of 1 : 0.4 : 1 (Shirasaka *et al*, 1996). Tegafur is an oral prodrug of 5-FU, which is gradually converted to 5-FU. 5-chloro-2,4-dihydroxy pyridine is a reversible inhibitor of dihydropyrimidine dehydrogenase, which catabolises 5-FU, leading to an increase in antitumour activity. Potassium oxonate is an orotate phosphoribosyltransferase inhibitor and decreases the incorporation of 5-FU triphosphate into RNA in the gastrointestinal mucosa. It reduces the incidence and severity of diarrhoea. According to the Japanese clinical trials of S-1, 80 mg m^−2^ day^−1^ was the recommended dose (RD). In phase II trials of S-1 against unresectable or recurrent gastric cancer, RRs were 44–49% with a low incidence of severe toxicities (Sakata *et al*, 1998; Koizumi *et al*, 2000).

CPT-11 is an inhibitor of DNA topoisomerase I. A Japanese late phase II study of CPT-11 as a single agent for AGC obtained an RR of 23% (Futatsuki *et al*, 1994). Some previous studies suggested a lack of crossresistance between CPT-11 with fluoropyrimidines (Houghton *et al*, 1996; Cao and Rustum, 2000). Combination of CPT-11 with 5-FU and leucovorin have shown promising activity not only in metastatic colorectal cancer but also in AGC (Douillard *et al*, 2000; Saltz *et al*, 2000; Assersohn *et al* 2004; Bouche *et al*, 2004; Pozzo *et al*, 2004).

We therefore conducted a phase I/II clinical study of combination treatment of S-1 with CPT-11. The primary objectives of the phase I study were to estimate the maximum-tolerated dose (MTD) of CPT-11 in combination with S-1 and to determine the RD for phase II studies. In the phase II study, we investigated the clinical activity and the feasibility of this chemotherapy regimen.

## PATIENTS AND METHODS

### Patients

Before entry, tumour size was determined by chest or gastrointestinal X-ray film, endoscopic examination of the upper gastrointestinal tract, computed tomographic (CT) scan of the abdomen, barium enema, and bone scintigram. Measurable lesions were selected according to response evaluation criteria in solid tumours (RECIST). A complete blood cell count, liver and renal function test, and urinalysis were performed within 7 days before entry. The eligibility criteria were as follows: age 20–80 years; histologically proven unresectable locally advanced or metastatic gastric adenocarcinoma; no previous chemotherapy or radiotherapy, adequate organ function, defined as haemoglobin >8 g dl^−1^, leucocyte count >4000–12 000 mm^−3^, platelet count >100 000 mm^−3^, serum bilirubin level <1.5 mg dl^−1^, serum transaminase (aspartate aminotransferase and alanine aminotransferase) <100 UI^−1^, alkaline phosphatase <twice the upper limit of the normal range (ULN), serum creatinine level less than the ULN; Eastern Cooperative Oncology performance status 0–1; expected survival period >3 months; oral intake of medicines is possible; and written informed consent from the patients. Patients with symptomatic brain metastases, large ascites, or pleural effusion were not eligible. This study was approved by the ethics committees in each institution.

### Treatment schedule

The fixed dose of S-1 (Taiho Pharmaceutical Co. Ltd, Tokyo, Japan) was 80 mg m^−2^ day^−1^. Three doses of S-1 were established according to body surface area (BSA) as follows: BSA <1.25 m^2^, 80 mg day^−1^; BSA 1.25–1.5 m^2^, 100 mg day^−1^; and BSA ⩾1.5 m^2^, 120 mg day^−1^, as described previously (Sakata *et al*, 1998; Koizumi *et al*, 2000). Patients received their assigned dose of S-1 divided in two, after breakfast and dinner orally. One course of therapy consisted of S-1 administered for 14 consecutive days. CPT-11 (Irinotecan; Daiichi Pharmaceutical Co. Ltd, Tokyo, Japan) was diluted in 500 ml physiological saline, and administered as a 90 min intravenous (i.v.) infusion on days 1 and 8. The starting dose of CPT-11 was 70 mg m^−2^ (level 1), which was to be increased in 10 mg m^−2^ increments to 100 mg m^−2^ (level 4), unless the MTD were achieved. No intrapatient dose escalation was allowed. At least three patients were treated at each dose level. If one of three patients at a given dose developed any dose-limiting toxicity (DLT), three or more patients were entered at the same dose. Before proceeding to the next dose level, all patients had received at least one course. This treatment course was repeated every 4 weeks with an allowance for a delay in treatment if toxicity was observed. The next course was started only for patients whose biological parameters had been maintained at levels satisfying the eligibility criteria, except for the leucocyte count (>3000 mm^−3^), and with no disease progression observed. Prophylactic administration of antiemetic medication (5-HT_3_ antagonist and corticosteroid) at standard doses was routinely used when CPT-11 was administered to prevent nausea and vomiting. The treatment was repeated unless disease progression or severe toxicity was observed.

### Evaluation

A complete blood cell count, liver and renal function test, and urinalysis were assessed at least once a week during the first course, and every other week afterwards. Before each course, additional examinations were performed to evaluate sites. The National Cancer Institute common toxicity criteria version 2.0 was applied to evaluate the toxicity of this therapy during each course. Dose-limiting toxicities were defined as grade 4 neutropenia, grade 4 thrombocytopenia, any febrile grade 3 or 4 haematological toxicity, or grade 3 nonhaematological toxicity (except nausea and vomiting) during the first course. The MTD was defined as the dose at which >33% patients experienced DLTs during the first course. Lesions noted at baseline and 1 week after each course were measured or evaluated by CT. Objective responses were evaluated according to the RECIST criteria. The survival period was calculated from the start of treatment to death or the latest followed-up day. The eligibility and suitability for assessment and the objective response to the treatment were reviewed extramurally.

## RESULT

Between January 2001 and December 2003, 51 eligible patients were entered in this study. The first 16 patients were entered into the phase I portion and the next 35 patients were entered into the phase II portion to confirm the toxicities and efficacy at the RD. All patients were eligible for toxicity evaluation in any course and objective response evaluations (Table 1). Thirty-one patients had undergone gastrectomy and none had received adjuvant chemotherapy after gastrectomy. Histological evaluation revealed 21 patients to be intestinal type and 30 patients to be diffuse type. A total of 267 courses were given. The median number of treatment courses was four (range: 1–16) and five (range: 1–13) in phase I and II portion, respectively (Table 2). The median duration of therapy per patient was 161 days (range: 28–637) in phase I portion, and 172 days (range: 28–599) in phase II portion, respectively. The median number of days until the start of the second course after completion of scheduled S-1 in the first course was 14 (range: 14–21 days) among 46 patients who were treated with two courses or more. Three of the six patients at level 3 and 4 required more than 14 days interval to start the second course, although none of 42 patients did in phase II portion.

### Determination of MTD

In the phase I portion at level 2, one patient developed grade 3 diarrhoea during the first course, but the other two patients in the same cohort showed no DLT. An additional four patients were enrolled for safety evaluation, but overall only one of the total of seven patients developed a DLT at 80 mg m^−2^ of CPT-11. As dose level 4, two of three patients exhibited DLTs in the first course, one of whom had grade 3 febrile leucopenia and neutropenia, and grade 4 thrombocytopenia, another had grade 3 nonhaematological toxicity (diarrhoea). The frequency of severe haematological and nonhaematological toxicities increased according to the increment of the CPT-11 dose (Table 3). Based on these results, dose level 4 was declared as the MTD, and level 3 should be declared as the initial RD according to the protocol. However, all three patients at level 3 experienced grade 4 haematological or grade 3 nonhaematological toxicities during the second course, followed by dose reduction of CPT-11 from the third course. The dose intensity per course of CPT-11 was 86% of planned CPT-11 dose at level 3, compared with 96% at level 2 (Table 2). Thus, considering the safety and the continuation of the treatment, the final RD was level 2 dose of 80 mg m^−2^ in the following phase II portion.

### Safety

In the 42 patients of the phase II portion including seven patients assigned at level 2 in the phase I portion, the most frequently observed severe (grades 3 and 4) haematological toxicity was neutropenia (6 cases, 14%) (Table 3). Frequently observed nonhaematological toxicities (all events) included nausea (25 cases, 59%), anorexia (23 cases, 55%), and vomiting (16 cases, 38%). In addition, the overall incidence of diarrhoea was 40% (17 out of 42); however, grade 3 or 4 diarrhoea was observed in four out of 42 (10%), and recovered within seven days (Table 3). During this study, eight patients received granulocyte colony-stimulating factor because of neutropenia. Incidences of the worst-grade toxicities in patients treated with the final RD were none (four cases, 10%), grade 1 (11 cases, 26%), grade 2 (16 cases, 38%), grade 3 (seven cases, 17%), and grade 4 (four cases, 10%), respectively. Neither treatment-related death nor delayed severe toxicity was observed.

### Efficacy

**Figure 1:**
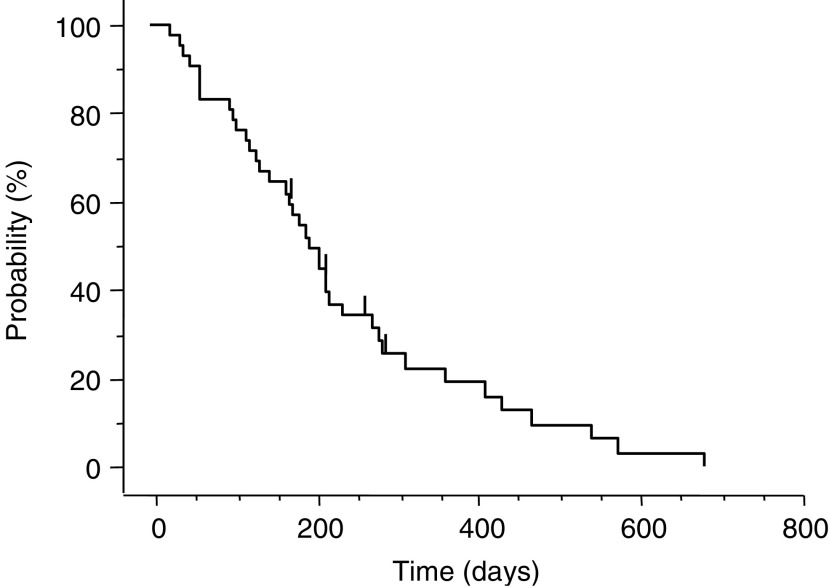
Time to progression curve for the 42 patients in the phase II portion; the median TTP was 195 days (range: 25–684 days).

**Figure 2:**
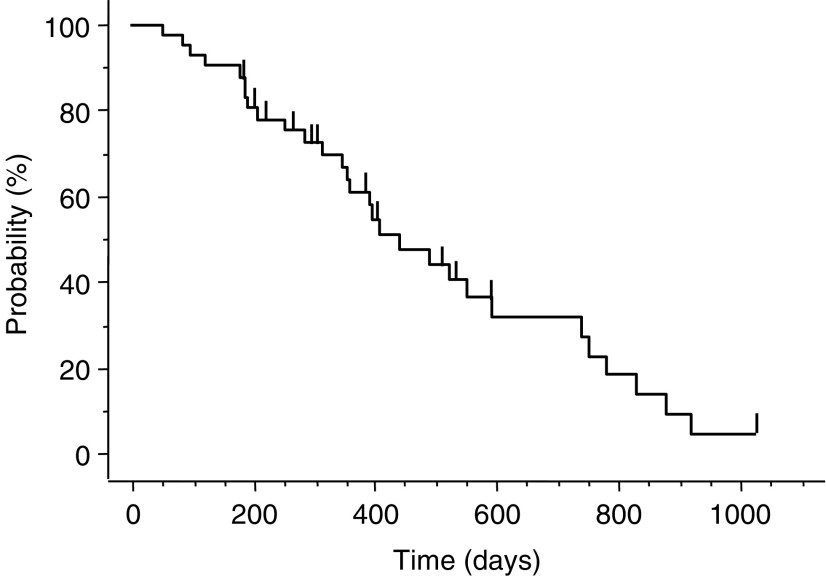
Cumulative overall survival for the 42 patients in the phase II portion. The median survival time was 444 days (range: 54–1029 days).

All 42 patients including seven patients assigned in phase I portion were evaluated to determine the RR at the RD. The RR at the RD in the phase II portion was 62% (26 of 42, 95% confidence interval (CI): 47.2–76.6%); 11 patients showed stable disease as their best response, five patients had PD (Table 4). The median time to progression (TTP) was 195 days (range: 25–684) in the phase II portion (Figure 1). The median time to response and the median overall durations of response in 26 responders in phase II portion were 48 (range: 28–158) and 178 days (range: 66–643), respectively. One patient was able to undergo gastrectomy after five courses of combination therapy. Subgroup analysis according to tumour lesion and pathological type for the 42 patients in phase II portion showed that the RR was 58% (seven of 12) for liver metastasis, 60% (15 of 25) for lymph node metastasis, 69% (nine of 13) for metastatic peritoneal nodule, and 69% (11 of 16) for primary lesions (Table 4), and the RR according to pathological type was 61% (11 of 18) for the intestinal type and 63% (15 of 24) for the diffuse type. The MST of patients in the phase II portion was 444 days (range: 54–1029) and 1- and 2-year survival rates were 61 and 28%, respectively (Figure 2). The median follow-up time for survival analysis was 736 days.

## DISCUSSION

This study was undertaken to determine the RD for a phase II study of CPT-11 combined with S-1 for metastatic advanced gastric cancer and to investigate the antitumour effect and feasibility of this combination. The RD was determined to be 80 mg m^−2^ of CPT-11 on day 1 and day 8, and 80 mg m^−2^ per day of S-1 on days 1–14 of a 28-day cycle. The phase II study using this combination obtained an RR of 62% (26 of 42), and in particular, the MST of 444 days and the TTP of 195 days for chemotherapy-naïve patients were promising. In addition, toxicity was mild and tolerable, and therapy was administrated on an outpatient basis.

Two late phase II studies of S-1 as a single agent in advanced gastric cancer in Japan obtained RRs of 44 and 49%, respectively (Sakata *et al*, 1998; Koizumi *et al*, 2000). Combined analysis of the results of these phase II studies suggested an MST of 244 days, while toxicities were generally mild. Based on these data, there are several ongoing combination studies of S-1 with another anticancer agent with a different mechanism of action, aimed at achieving more survival benefit.

CPT-11 was shown to lack crossresistance with fluoropyrimidines in both experimental and clinical settings (Vanhoefer *et al*, 2001). The response rate of CPT-11 alone in gastric cancer was 23% in a Japanese phase II study (Futatsuki *et al*, 1994). The response rate in patients with previous 5-FU-containing regimens was 18.9%, which indicated a lack of crossresistance between CPT-11 and 5-FU in gastric cancer. Preclinical studies of human cancer cell lines and tumour xenografts have suggested that the combination of CPT-11 and 5-FU has additive-to-synergistic antitumour activities (Houghton *et al*, 1996). Thus, we selected CPT-11 as the combination agent to be used with S-1.

When CPT-11 is combined with S-1, there is concern about the increase of the frequency of severe diarrhoea, which is the common toxicity not only in CPT-11 alone regimen but also in S-1 alone regimen. As the median time to deteriorate into the worst grade of S-1-induced diarrhoea was 15 days of consecutive S-1-alone administration (Nagashima *et al*, 2005), we planned that CPT-11 was administrated i.v. on day 1 and day 8, and S-1 was orally taken for 2 consecutive weeks followed by 2-week drug holiday. In this phase I portion, S-1 was given at a fixed dose of 80 mg m^−2^ day^−1^ and the CPT-11 dose was escalated from 70 mg m^−2^ as level 1 to 100 mg m^−2^ as level 4. DLTs were observed in two of three patients at level 4, which was defined as the MTD. DLTs consisted of grade 3 febrile neutropenia, grade 4 thrombocytopenia, and grade 3 diarrhoea. According to the protocol conditions, the initial RD of CPT-11 combined with S-1 should be the level 3 dose of 90 mg m^−2^; however, all three patients assigned the level 3 dose experienced grade 4 haematological or grade 3 nonhaematological toxicities in the second course. Considering the safety and the ability to continue treatment, the final RD of CPT-11 was the level 2 dose of 80 mg m^−2^. In the phase II portion, the incidence of the most common toxicities (grade 3 or 4) was 14% for neutropenia, and 10% for diarrhoea and anorexia. Thus, mild and tolerable toxicities resulted in the median treatment course of 5, achieving an RR of 62 and MST of 444 days.

Three other phase I studies for combination therapy with CPT-11 and S-1 have been reported. Yamada *et al* (2003) reported an RD of 150 mg m^−2^ CPT-11 administration on day 1 with 80 mg m^−2^ day^−1^ S-1 administration from day 1 to day 14 of a 21-day cycle. Another study assigned patients to receive 80 mg m^−2^ CPT-11 on days 1 and 15, and S-1 from day 1 to day 21, followed by a 2-week rest (Takiuchi *et al*, 2005). In the two regimens, the doses of S-1 were similar to single-agent therapy of S-1, which consist of 4-week consecutive administration with a 2-week rest. On the other hand, Komatsu *et al* (2005) reported the regimen of 125 mg m^−2^ CPT-11 on day 1 and day 15, combined with 80 mg m^−2^ day^−1^ S-1 administration from day 1 to day 14 of a 28-day cycle. The dose intensity of S-1 in that regimen, as in ours, is smaller than that of the single-agent S-1 therapy. Little constructive information can be obtained by comparing the results of these different studies. However, we are the first to report the promising result of the phase II portion for combination therapy with CPT-11 and S-1, in detail.

Previous reports indicated that 5-FU might inhibit the conversion of CPT-11 to SN-38, which is its active form (Sasaki *et al*, 1994; Falcone *et al*, 2001). On the contrary, it has been reported that pharmacokinetic (PK) analysis of CPT-11 when combined with S-1 showed no change in any PK parameter as compared with the expected values for CPT-11 as a single agent (Yamada *et al*, 2003). Additionally, the PK results of S-1 combined with CPT-11 were similar to those obtained by S-1 single-agent treatment (Takiuchi *et al*, 2005). Taken together with these data, it appears there is no PK interaction between CPT-11 and S-1.

Cisplatin has been employed in the treatment of AGC. Boku *et al* (1999) reported the promising results of 48% RR and 322 days MST for AGC treated by CPT-11 combined with CDDP, with acceptable toxicity. A phase I/II study of S-1 combined with CDDP indicated the surprising results of an RR of 74% (Koizumi *et al*, 2003). However, the MST of S-1 combined with CDDP was 383 days, which was shorter than the 444 days in our study. The incidence of grade 3 or 4 haematological toxicities was almost the same (16 and 19% in the CDDP combination and CPT-11 combination, respectively), whereas the incidence of nonhaematological toxicities was 26% in S-1 combined with CDDP, which was higher than the 10% in our study. Additionally, in the combination of CDDP, it is necessary to hydrate patients with drip infusion to avoid of CDDP-induced renal damages. Thus, S-1 combined with CPT-11 might be less toxic and more easily manageable in outpatient clinics than the CDDP-combined regimens.

In three randomised phase II trials, the combination of CPT-11 and 5-FU/LV was compared with the combination of CPT-11 and CDDP (Pozzo *et al*, 2004), CDDP and 5-FU/LV (Bouche *et al*, 2004), and etoposide and 5-FU/LV (ELF) (Moehler *et al*, 2005). All three trials indicated that the combination of CPT-11 and 5-FU/LV was the most effective combination and will be assessed in a phase III trails. The RR, TTP, and MST in the combination arm of CPT-11 and 5-FU/LV ranged from 30 to 40%, 4.5–6.9 months, and 10.8–11.3 months, respectively, with acceptable toxicity profiles. In addition, the randomised phase III study confirmed that the combination of CPT-11 and 5-FU/LV is superior in terms of TTP, compared with the combination of CPT-11 and CDDP (Dank *et al*, 2005). These results underline the potential role of the combination of CPT-11 and 5-FU/LV. If S-1 could be used instead of 5-FU infusion, S-1 combined with CPT-11 might become an alternative to the combination of CPT-11 and 5-FU/LV. We are awaiting the results of a randomised phase III trial (5-FU infusion *vs* S-1 *vs* CPT-1 with CDDP) for AGC patients.

In conclusion, our phase I/II trial showed S-1 combined with CPT-11 is effective and well tolerated with acceptable toxicity. This regimen should be one of the choices for an experimental arm in phase III trials in near future.

## Figures and Tables

**Table 1:** Patient characteristics

	**Phase I portion**				**Phase II portion**
**Level**	**1**	**2**	**3**	**4**	
**CPT-11 (mg m^−2^)**	**70**	**80**	**90**	**100**	**80**
**No. of patients**	**3**	**7**	**3**	**3**	**35**
*Age (years)*					
Median	70	57	58	63	63
Range	68–76	39–73	51–77	21–67	47–79
<65	0	5	2	2	18
⩾65	3	2	1	1	17
					
*Sex*					
Female	0	1	0	1	13
Male	3	6	3	2	22
					
*Pathology*					
Intestinal	2	2	1	0	16
Diffuse	1	5	2	3	19
					
Gastrectomy	1	5	2	2	21

**Table 2:** Completed course and dose intensity (DI)

	**Phase I portion**				**Phase II portion^a^**
**Level**	**1**	**2**	**3**	**4**	
**CPT-11 (mg m^−2^)**	**70**	**80**	**90**	**100**	
***n***	**3**	**7**	**3**	**3**	**42**
*Course*					
Median	9	4	6	3	5
Range	6–16	1–13	3–7	2–4	1–13
					
*S-1*					
DI	1120	1120	1120	1089	1099
%DI	100	100	100	97	98
					
*CPT-11*					
DI	140	155	155	169	151
%DI	100	96	86	84	94

DI=dose (per m^2^) per course.

a Including seven patients at level 2 of phase 1 portion.

**Table 3:** Toxicity incidence

**Course**	**Phase I portion** **First course**								**Phase II portion^a^** **All courses**	
		
**CPT-11 (mg m^−2^)**	**70**		**80**		**90**		**100**		**80**	
**No.of patients**	**3**		**7**		**3**		**3**		**42**	
**Toxicity/grade**	**All events**	**Grade 3/4**	**All events**	**Grade 3/4**	**All events**	**Grade 3/4**	**All events**	**Grade 3/4**	**All events**	**Grade 3/4**
*Haematological*										
Leucopenia	1	0	3	0	1	0	3	1	24	5
Neutropenia	0	0	3	0	1	0	3	1	20	6
Anaemia	1	0	0	0	2	0	2	1	20	3
Thrombocytopenia	0	0	0	0	0	0	1	1	5	2
										
*Nonhaematological*										
Anorexia	3	0	3	0	2	0	2	1	23	4
Nausea	3	0	4	0	2	0	2	1	25	3
Vomiting	0	0	1	0	2	0	1	1	16	2
Diarrhoea	0	0	1	1	1	0	1	1	17	4

a Including seven patients at level 2 of phase 1 portion.

**Table 4:** Response rate

	***n***	**CR**	**PR**	**SD**	**PD**	**Response (%)**
Phase II portion	42	0	26	11	5	62
Lymph nodes	25	0	15	8	2	60
Liver	12	0	7	3	2	58
Peritoneum	13	0	9	2	2	69
Primary	16	0	11	3	2	69
						
Total	51	1	30	15	5	61

CR=complete response; PR=partial response; SD=stable disease; PD=progressive disease.

Response rate=number of CR and PR/total number (*n*).
